# Robust Chiral High‐*Q* Response Driven by Quasi‐Bound Flatband in the Continuum

**DOI:** 10.1002/nap2.70218

**Published:** 2026-07-21

**Authors:** Lei Huang, Weiqing Wang, Han Shen, Jing Chen, Shifeng Li, Biao Liu, Fuhuan Shen

**Affiliations:** ^1^ State Key Laboratory of Flexible Electronics College of Chemistry and Life Sciences, College of Electronic and Optical Engineering Nanjing University of Posts and Telecommunications Nanjing China; ^2^ National Laboratory of Solid State Microstructures College of Engineering and Applied Sciences Nanjing University Nanjing China; ^3^ Division of Physics and Applied Physics School of Physical and Mathematical Sciences Nanyang Technological University Singapore; ^4^ School of Physics Central South University Changsha Hunan China

**Keywords:** chiral bound states in the continuum, guide mode, light‐matter interaction, photonics flatband

## Abstract

Chiral bound states in the continuum (BICs) with infinite radiation quality factor (*Q*‐factor) and topological robustness have provided a new paradigm for enhancing chiral light–matter interaction. However, the strong momentum‐space dispersion leads to a rapid collapse of the *Q*‐factor when the resonance deviates from the ideal BICs condition, severely limiting its practical applications. Here, by strategically engineering the interaction among four counterpropagating folded guided modes, we theoretically propose and numerically realize the chiral flatband quasi‐BICs. The pronounced chiroptical response arises from the destructive interference of two antiphase oscillating intrinsic modes, whereas the flatband characteristic originates from the interactions among opposite curvature hybrid guided modes. Compared with conventional BICs featuring strongly dispersive bands, our dispersionless BICs provide stronger light field confinement, enabling highly efficient third‐harmonic generation (conversion efficiency up to 2.3 × 10^−3^ under a pump intensity of 1 MW/cm^−2^). Our work establishes a new benchmark for miniaturized nonlinear chiral sources and may pave the way for the development of novel chiral optical device.

## Graphical abstract

This work theoretically proposes and numerically realizes chiral flatband quasi‐BICs by engineering four counterpropagating folded guided modes. The chiroptical response arises from destructive interference of two antiphase intrinsic modes, and the flatband originates from the interactions among opposite curvature hybrid guided modes. These dispersionless BICs provide stronger field confinement than conventional BICs, enabling highly efficient third‐harmonic generation, paving the way for miniaturized nonlinear chiral optical devices.

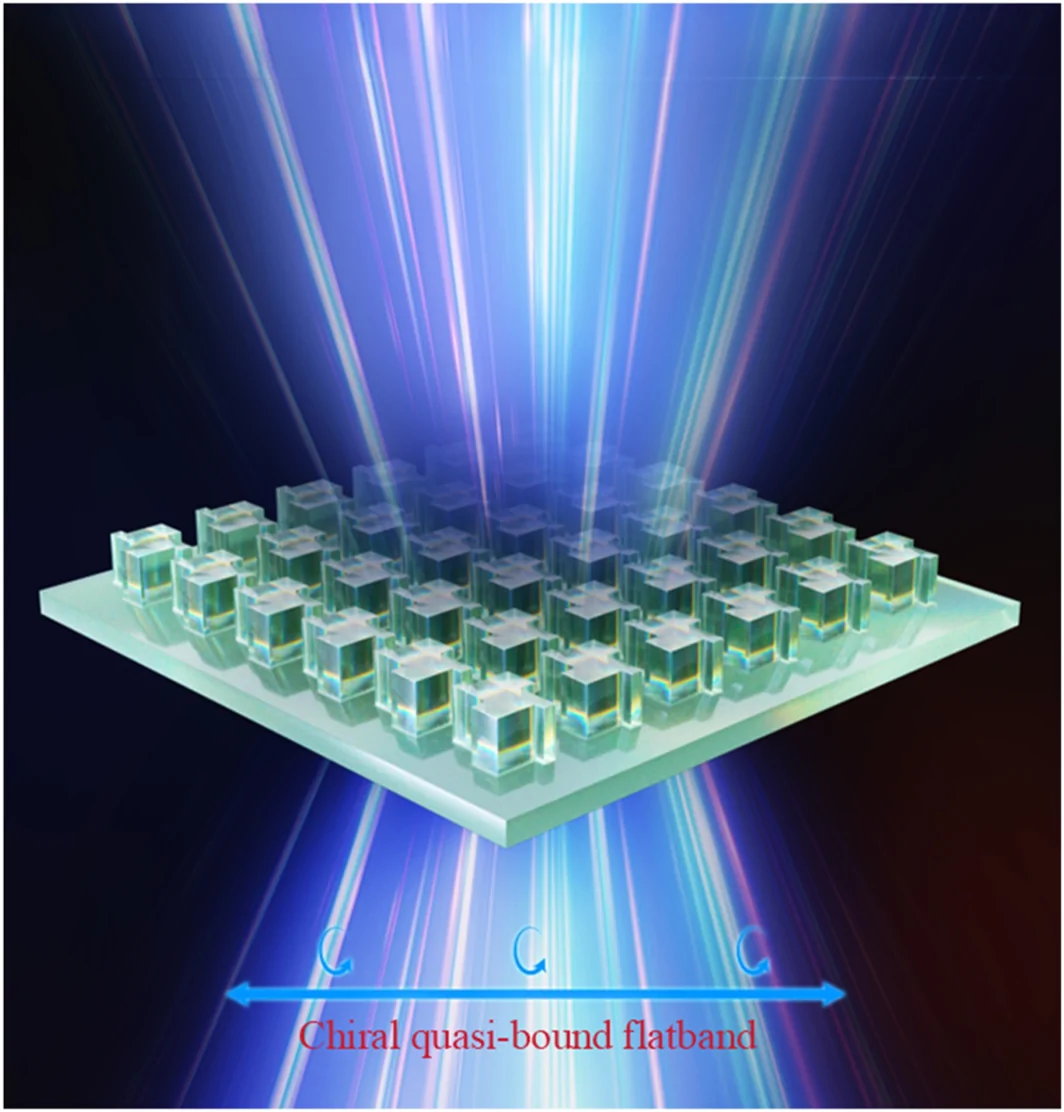

## 1 Introduction

Chirality, arising from the breaking of mirror or rotational symmetry, is a basic property in nature with significant implications across multiple scientific and technological fields [1, 2]. In optics, chiral phenomena originate from the selective interaction of a chiral object with the spin angular momentum of photons and are quantitatively characterized by circular dichroism (CD) spectroscopy [3, 4]. In general, the CD response in natural materials is weak due to the scale mismatch between chiral molecules and optical wavelengths [5]. Artificially engineered chiral metasurfaces have been developed to enhance these weak chiroptical signals. However, their practical application in chiral optics remains limited by significant ohmic and scattering losses within supported optical modes, which typically result in a low‐quality factor (*Q*‐factor) [6].

Bound states in the continuum (BICs) are nonradiative eigenmodes embedded inside the radiation continuum, which emerges as Bloch resonances in periodic lattice structure, and have been demonstrated as a revolutionary solution for the significant problems of chiral photonics [7, 8, 9, 10, 11]. Despite possessing an infinite radiation lifetime, the applications of BICs remain limited due to their intrinsic angle‐dependent dispersion, which comes with a rapid collapse of the *Q*‐factor that is unavoidable during fabrication and measurement processes [12]. Merging multiple BICs in momentum space to form super‐BICs offers a feasible approach to alter the scaling rule and boost *Q*‐factors as coupling among multiple BICs around a single BIC effectively reduces radiation and scattering losses [13, 14]. However, to achieve further control over light–matter interaction and enhance *Q*‐factors, novel physical mechanisms still need to be explored in BICs research. Engineering the energy‐momentum dispersion of photonic structures is at the heart of contemporary optics research. Among these, the weakly dispersive or even dispersionless (flat) bands have garnered significant attention because their vanishing group velocity and high density of states (DOS) can significantly enhance light–matter interactions and reduce radiation or scattering loss, offering great potential for applications in nonlinear optics and laser [15, 16, 17, 18, 19]. Recently, moiré superlattice and disorder‐induced band‐folding approaches have been proposed to achieve flatband BICs, but their experimental fabrication remains challenging [20, 21], and the associated chiral or nonlinear properties, as well as the underlying mechanisms, have not yet been thoroughly investigated.

Here, by leveraging the interaction among four counterpropagating folded guided modes, we achieve the nondispersive high‐*Q* chiral quasi‐BICs over a wide incident angular range of ± 5° (*Q*‐factor exceeding 20,000 and circular dichroism > 0.6). The strong chiral response is demonstrated to arise from the intrinsic destructive interference of two antiphase oscillating polarization modes. While the flatband is revealed to originate from the interactions among opposite curvature hybrid guided modes. Furthermore, leveraging these unique field confinement characteristics of the flatband BICs, we demonstrate highly efficient chiral third‐harmonic generation (THG) with conversion efficiencies up to 2.3 × 10^−3^ under a pump intensity of 1 MW/cm^−2^. This work opens a new route toward practical chiral nanophotonic devices, with promising applications in chiral lasing and advanced nonlinear polarization imaging.

## 2 Results and Discussion

Figure 1a depicts the schematic diagram of our designed flatband chiral metasurface, with its detailed geometry parameters illustrated in the inset (see more information in caption). A chiral cross metasurface with *C*_2_ rotational symmetry but no in‐plane mirror symmetry is constructed onto a SiO_2_ substrate. The calculated band structure and *Q*‐factor in *k*‐space are presented in Figure 1b,c. Three eigenmodes with distinct properties are identified within the investigated energy range. Both modes 1 and 2 exhibit divergently high *Q*‐factors at the *Γ*‐point, confirming their nature as symmetry‐protected BICs (SP‐BICs). In contrast, the *Q*‐factor of mode 3 remains nearly constant with increasing in‐plane wavevector (*k*_x_), identifying it as a guided mode. Besides, compared with two emerged BICs modes, mode 2 exhibits a higher *Q*‐factor with slower decay rate than mode 1, which indicates they have intrinsically distinct radiation decay nature.

**FIGURE 1:**
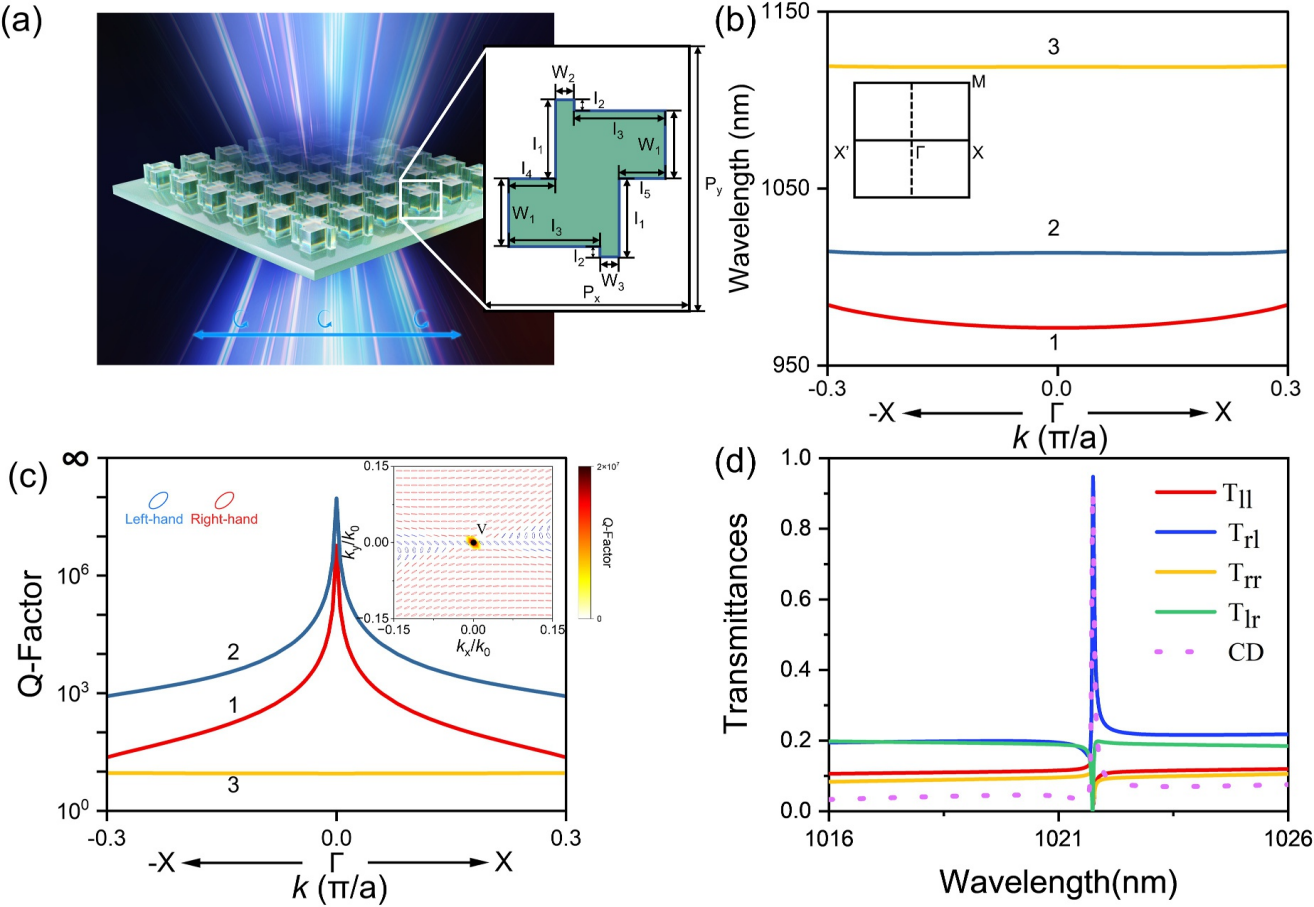
(a) Schematic of the metasurface with its detailed geometry parameters illustrated in inset. Here, the periods *P*_*x*_ = 485 nm and *P*_*y*_ = 600 nm, the height *H* = 260 nm, and the width and length are specified as follows: *W*_1_ = 160 nm, *W*_2_ = *W*_3_ = 45 nm, *L*_1_ = 185 nm, *L*_2_ = 25 nm, *L*_3_ = 215 nm, and *L*_4_ = 115 nm. In the simulation, the periodic boundary conditions are employed in the *x*‐, *y*‐directions, and the perfectly matched layer is set along *z*‐direction, and the refractive index of Si and SiO_2_ are chosen as 3.4 and 1.45, respectively [22]. (b, c) Simulated band structure and *Q*‐factors of the eigenmodes in the *k*‐space. Insets of Figure 1c show the eigenpolarization maps with the *Q*‐factor distribution for mode 2. (d) Calculated transmission and CD spectra of the chiral metasurface with *α* = 10 nm under different circular polarization incidences.

The far‐field eigenpolarization maps with the *Q*‐factor distribution for mode 2 are given in the inset of Figure 1c. Both an ultrahigh *Q*‐factor and a polarization singularity (*V*‐point) emerge at the *Γ* point, further confirming that mode 2 is an SP‐BIC. Additionally, the chiral property of mode 2 is demonstrated by the far‐field eigenpolarization map in Figure S1 (Supporting Information S1: Note 1), where breaking the *C*_2_ symmetry causes the *V* point to split into two *C* points away from the *Γ* point, giving rise to a chiral quasi‐BIC with nonvanishing helicity. The simulated transmission spectra for different polarization components *T*_*ij*_ under LCP and RCP normal illumination for mode 2 at *α* = 10 nm are shown in Figure 1d. The subscripts “*i*” and “*j* ”in *T*_*ij*_ represent the polarization states of the transmitted and incident light, respectively. An ultranarrow band chiral resonance with transmittance peak *T*_rl_ = 0.95 and transmittance dip *T*_rr_ = *T*_lr_ = *T*_ll_ ≈ 0 are observed at the wavelength of 1022 nm, which demonstrates the chiral quasi‐BICs characteristics. Besides, the emerged chiral quasi‐BICs possess an ultrahigh *Q*‐factor of ∼20,000 with a CD peak of 0.91 under the large asymmetry parameter (*α* = 10 nm), derived from *Q* = *λ*_0_/Δ*λ* and $\text{CD}=\frac{\left(T_{rr}+T_{lr}\right)-\left(T_{ll}+T_{rl}\right)}{\left(T_{rr}+T_{lr}\right)+\left(T_{ll}+T_{rl}\right)}$ (*λ*_0_ is the resonance wavelength, Δ*λ* is the full width at half maximum), which confirms the intrinsically low radiation decay nature of the quasi‐BICs.

To reveal the underlying physics origins of the high‐*Q* response, the zoomed‐angle‐resolved LCP and RCP transmission and CD spectra are simulated in Figure 2a–f. Here, for mode 2, a flatband dispersion with a missing point at the normal direction (*Γ* point) supported by the SP‐BICs is further demonstrated in the transmission or CD as shown in Figure 2d–f, and comparing with the parabolic‐like energy band presented in mode 1 (shown in Figure 2a–c), the dispersionless behavior constitutes the physical basis for the high‐*Q* response resulting from the reduced radiation and scattering loss. To clearly explain these reasons, we calculate and compare the magnetic field distributions of mode 1 and mode 2 at their respective resonance wavelengths with the same *α* = 10 nm as shown in Figure 2i,j, respectively. It is clearly seen that mode 1 is an electric dipolar resonance, which possesses a weak optical field localization, thus resulting in the strong scattering and radiation loss at the edge of configuration. Although mode 2 is a typically magnetic dipolar mode, exhibiting a strong optical field localization within the configuration due to the nearly zero group velocity and infinitely large effective mass, and thus results in the low radiation and scattering loss at the edge of configuration. The further quantitative transmission and CD spectra of mode 1 and 2 at their respective resonance wavelengths with the same *α* = 10 nm are given in Figure 2g,h. Obviously, in contrast to strong dispersion for mode 1, the flatband BICs exhibits a much narrower linewidth and higher *Q*‐factor. More specifically, the chiral flatband response of mode 2 is further verified by calculating the transmission spectra over a wide incident angular range of sin(*θ*) from −0.08 to +0.08 as shown in Supporting Information S1: Note 2.

**FIGURE 2:**
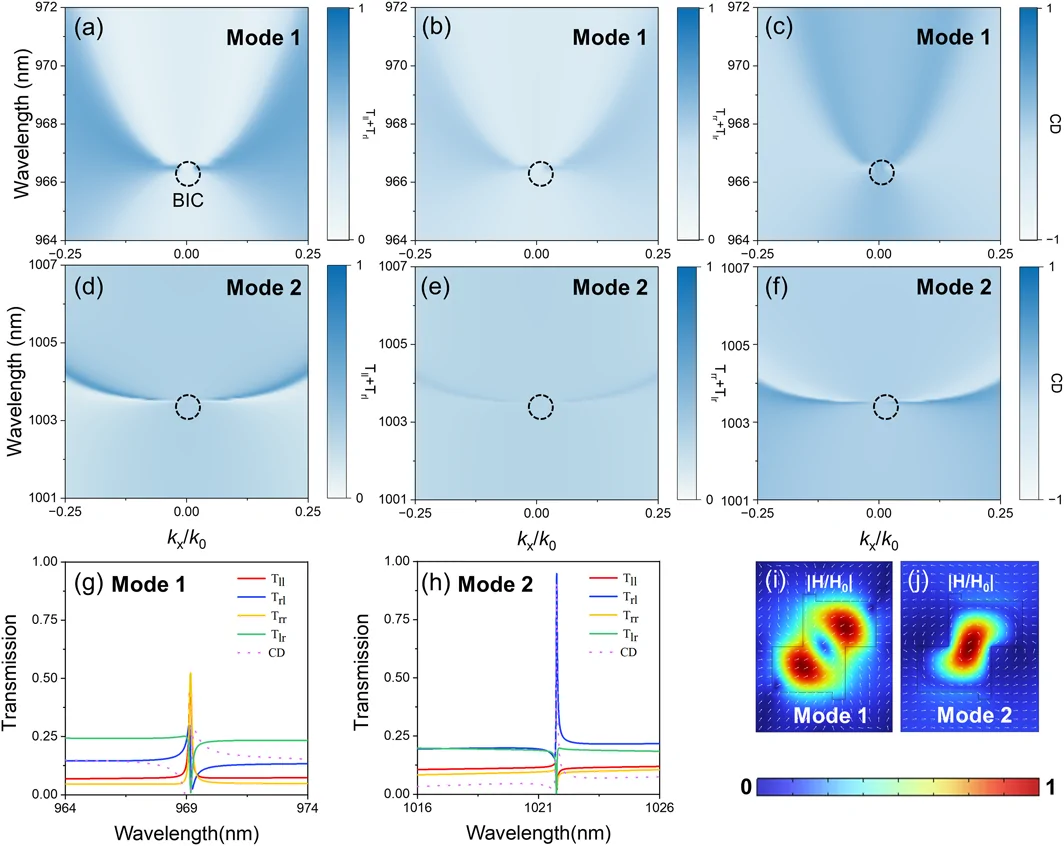
(a–c) are the calculated angle‐resolved transmission and CD spectra under LCP and RCP incidence with the *α* = 0 for mode 1, where SP‐BICs emerge at the *Γ*‐point (marked by circular dash line). Panels (d–f) are the calculated angle‐resolved transmission and CD spectra under LCP and RCP incidences with the *α* = 0 for mode 2, where the flatband BICs emerge at the *Γ*‐point (marked by the dashed line). (g, h) The quantitative transmission and CD spectra at normal incidence with the *α* = 10 nm for mode 1 and mode 2, respectively. Panels (i, j) show the calculated normalized magnetic field distributions for modes 1 and 2, where the different magnetic field profile demonstrates the formation mechanism of two modes.

To elaborate on the mechanism behind flatband dispersion, the coupling dispersion equation between the four lowest‐energy folded guided modes is considered and written as follows [23, 24]:

(1) $$\omega_{1}\left(q\right)=\frac{1}{n_{x}}+\frac{1}{n_{g}}\left(\sqrt{{\left(1+q_{x}\right)}^{2}+q_{y}^{2}}-1\right)$$

(2) $$\omega_{2}\left(q\right)=\frac{1}{n_{x}}+\frac{1}{n_{g}}\left(\sqrt{{\left(-1+q_{x}\right)}^{2}+q_{y}^{2}}-1\right)$$

(3) $$\omega_{3}\left(q\right)=\frac{1}{an_{y}}+\frac{1}{n_{g}}\left(\sqrt{q_{x}^{2}+{\left(\frac{1}{a}+q_{y}\right)}^{2}}-\frac{1}{\alpha }\right)$$

(4) $$\omega_{4}\left(q\right)=\frac{1}{an_{y}}+\frac{1}{n_{g}}\left(\sqrt{q_{x}^{2}+{\left(-\frac{1}{a}+q_{y}\right)}^{2}}-\frac{1}{\alpha }\right)$$

where *q*_x_ = *k*_x_·*P*_x_/2π and *q*_y_ = *k*_y_·*P*_y_/2π are the in‐plane wavevectors, *n*_*x*_, *n*_*y*_ are effective refractive indices of guided modes propagating along *x* and *y*‐direction, *n*_*g*_ is the group refractive index, and *a* = *P*_y_/*P*_x_ is the anisotropic factor, which is variable with the period *P*_y_. The interplay of diffractive and radiative coupling among these four folded guided modes determines the curvature of the bands. In particular, the hybridization of the positive‐curvature states at off‐*Γ* points and the negative‐curvature state at the *Γ*‐point, leads to the formation of flatband. To clearly present the evolution process, the geometric configurations of the metasurface with different *y*‐directional periods and their corresponding band diagrams, along with the coupling dispersion curves for the four lowest‐energy folded guided modes (n, *m* = ± 1, 0 and *n*, *m* = 0, ± 1), are shown in Figure 3a–j. Initially, when *P*_x_ = *P*_y_ = 485 nm (Figure 3a,f), mode 2 exhibits a typical parabolic dispersion with a positive‐curvature of BICs at the *Γ*‐point. Here, the formation of mode 2 band results from the interaction of the four counterpropagating guided modes, and the curvature is determined by the positive‐curvature states at off‐*Γ* points and the negative‐curvature state at the *Γ*‐point as indicated in Figure 3f. As *P*_y_ increases, the dispersion curvature of mode 2 progressively decreases; meanwhile, the folded guided modes along the *y*‐direction (*n*, *m* = 0, ± 1) are red‐shifted (Figure 3g–i). When the *P*_y_ reaches 600 nm (Figure 3j), the dispersion curvature approaches zero. The red‐shifted guided modes along the *y*‐direction modulate the negative‐curvature state at off‐*Γ* points and ultimately hybridize with the positive‐curvature state at *Γ* points, leading to the formation of flatband [24, 25]. The variation of band curvature with periods is quantified in Figure 3k.

**FIGURE 3:**
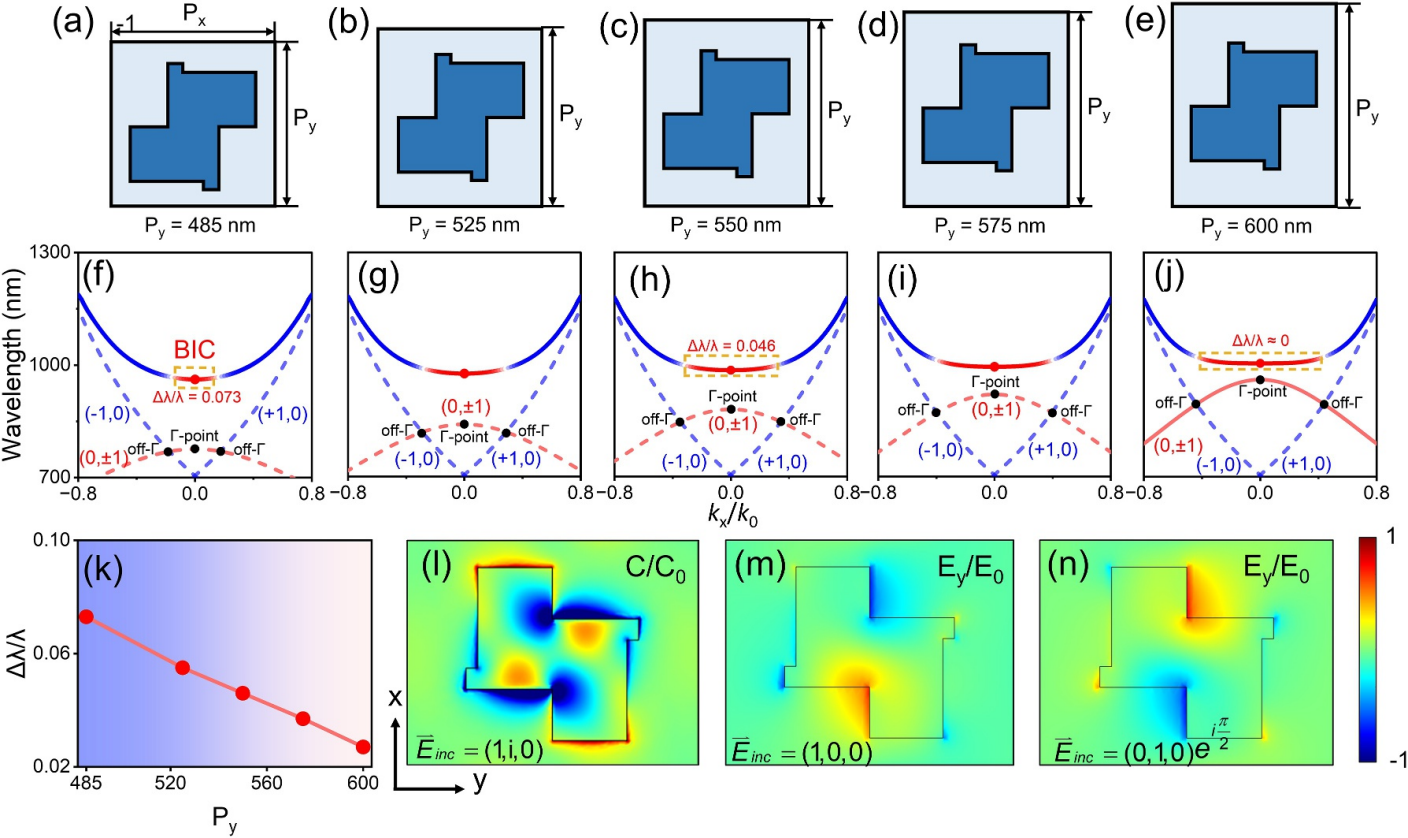
(a–e) Schematic diagrams of the chiral flatband metasurface with *P*_y_ = 485, 525, 550, 575, and 600 nm, respectively, where *P*_x_ is fixed. (f–j) Photonic band diagrams along the *k*_x_ direction for the corresponding structures in panels (a–e). The counterpropagating guided modes along the *x‐*direction are labeled as (± 1, 0) (blue solid lines), whereas the degenerate guided modes along the *y‐*direction are labeled as (0, ± 1) (red dashed lines). The corresponding curvatures are labeled in the figures. The hybridization of the positive‐curvature states at off‐*Γ* points and the negative‐curvature state at the *Γ*‐point, leads to the formation of flatband. (k) Band curvature Δλ/λ of the flatband BICs as a function of the lattice period *P*_y_. (l) Normalized optical chirality field distribution for the flatband BICs at *Γ*‐point. (m, n) Normalized in‐plane *E*_y_ distributions under (m) *x*‐polarized and (n) *y*‐polarized excitation at the resonance wavelength 1015 nm with a π/2 phase difference.

To further investigate the chiral origin and near‐field enhancement feature, we calculate the near‐field optical chirality distributions and the in‐plane *E*_y_ distributions under the *x* and *y* polarization incidences with π/2 phase differences as shown in Figure 3i–n, respectively. Here, the optical chirality emerged at arbitrary point of space is quantitatively estimated based on the superposition between the complex electric field ${\vec{E}}^{*}$ and magnetic field $\vec{B}$ as follows [26, 27]:

(5) $$C\left(\vec{r}\right)=-\frac{\varepsilon_{0}\cdot \omega }{2}\text{Im}\left[{\vec{E}}^{*}\left(\vec{r}\right)\cdot \vec{B}\left(\vec{r}\right)\right]$$

where *ε*_0_ and *ω* are the permittivity of vacuum and the angular frequency, respectively. The pronounced and inhomogeneous sign distribution in the calculated near‐field optical chirality (Figure 3l) indicates strong chiral enhancement potential for far‐field and optical nonlinearity. Additionally, it is possible to reveal the underlying chirality origin by calculating the electric field distributions using two orthogonal linearly polarized light excitations with a ± π/2 phase difference, since circularly polarized light can be understood as being composed of two orthogonal linearly polarized light beams with equal amplitude and a ± π/2 phase difference. Figure 3m,n show the opposite *E*_y_ phase distributions under orthogonal linear polarization incidences with a + π/2 phase difference, confirming that the observed chirality results from destructive interference between the two intrinsic linear polarization modes.

Figure 4a shows the calculated results of mode volume and field enhancement factor for the dispersive and nondispersive BICs. The effective mode volume *V*_mode_ is obtained by integrating the energy density *W* over the space on cross structures, and normalizing it to the maximum value of *W* at each wavelength: $V_{\text{mode}}\left(\lambda \right)=\frac{\int W\left(r,\lambda \right)d^{3}r}{\max\left[W\left(r,\lambda \right)\right]}$ [28]. Clearly, the nondispersive BICs exhibit stronger localized field and smaller mode volume, which is expected to possess great potential for chiral nonlinear generation and conversion, since the nonlinear conversion efficiency scales with *V*_mode_ according to the equation ${\left(\frac{Q}{V_{\text{mode}}}\right)}^{2}\cdot I_{\text{pump}}^{2}$, where *I*_pump_ is pump intensity [29]. Figure 4b presents the simulated THG spectra for the two BIC modes. As expected from theory predictions, the THG signal from the nondispersive BICs is pronouncedly stronger than that from the conventional dispersive BICs, and the nonlinear conversion efficiency is evaluated to be 2.3 × 10^−3^ under a pump intensity of 1 MW/cm^2^, based on the equation: $\eta_{\text{THG}}=\frac{P_{\text{THG}}}{P_{\text{FW}}}$ (where *P*_THG_ and *P*_FW_ are the radiated power of the third harmonic wave and fundamental wave, respectively). It should be noted that, for practical experimental measurements, the actual conversion efficiency is lower than the calculated value due to material absorption, fabrication imperfections, and thermal effects. The log‐log plot of emission power as a function of pump power is further given in Figure 4c. Linear fitting yields slopes close to 3 for both modes, confirming the cubic power scaling expected for THG. Finally, to more clearly highlight the unique advantages of our work compared with previous studies, we have added Table 1, which summarizes the flatband and nonflatband characteristics, together with the corresponding *Q*‐factors and CD values reported in recent years. Here, the flatband is defined based on the band curvature, quantified by Δ*λ*/*λ*. When Δ*λ*/*λ* is close to zero, such that the wavelength variation within the investigated angular range can be neglected, the band is classified as a flatband. Otherwise, it is classified as a nonflatband. This comparison clearly reveals that our flatband BICs design simultaneously achieves robust high *Q*‐factors and strong CD across a wide angular range, outperforming most previously reported investigations.

**FIGURE 4:**
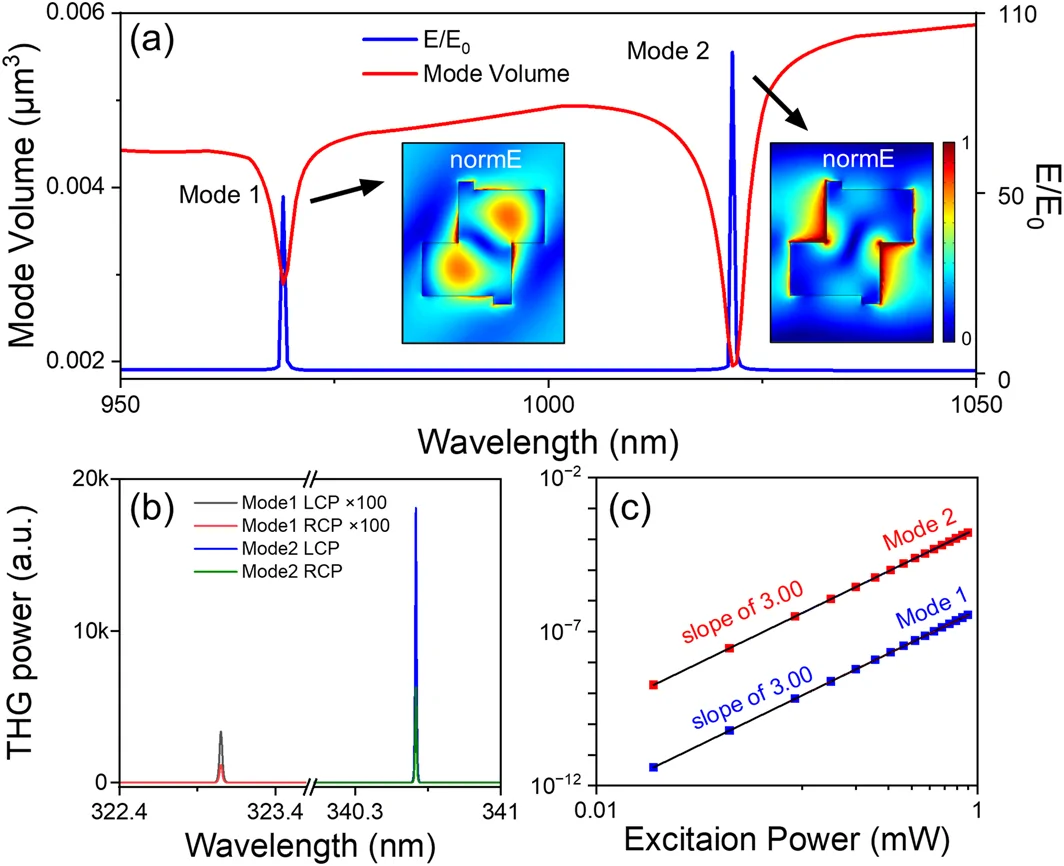
(a) Calculated mode volume and normalized electric field intensity as a function of wavelength. The insets show the calculated normalized electric field distributions for mode 1 and mode 2, respectively. (b) Simulated THG spectra under different circularly polarized light incidences. Obviously, the flatband BICs can induce much stronger THG signal than the conventional BICs mode. (c) The dependence of the THG intensity from modes 1 and 2 on the excitation intensity is plotted in log‐log scale. The cubic linear fit is displayed by the black line.

**TABLE 1:** Performance comparisons of the *Q*‐factor and CD in our investigated flatband BICs with the conventional chiral BICs reported in the past few years [27, 30, 31, 32, 33, 34, 35, 36, 37, 38, 39, 40].

Work details	Simulated/measured	CD	*Q*‐factor	Dispersion feature	Flatband range (°)
*Nat*. *Commun*. 2022, 13 (1): 4111 [30].	Simulated/measured	0.95 (simulated)	261 (simulated)	Non‐flatband	None
		0.83 (measured)	142 (measured)		
*Science*, 2022, 377 (6611): 1215–1218 [31].	Simulated/measured	0.99 (simulated)	40,000 (simulated)	Non‐flatband	None
		0.92 (measured)	5000 (measured)		
*Nature*, 2023, 613 (7944), 474–478 [32].	Measured	0.93	2663	Non‐flatband	None
*Phys*. *Rev*. *B*, 2023, 108 (15), 155,410 [33].	Simulated	0.95	482	Non‐flatband	None
*Adv*. *Opt*. *Mater*. 2024, 12 (16), 2303195 [34].	Simulated	0.73	5900	Non‐flatband	None
*Opt*. *Lett*. 2024, 49 (20), 5703 [35].	Simulated	0.95	6137	Non‐flatband	None
*Phys*. *Rev*. *Lett*. 2025, 134 (1), 013,805 [36].	Simulated	0.99	120,000	Non‐flatband	None
*Phys*. *Rev*. *Lett*. 2025, 134 (10), 103,801 [27].	Measured	0.93 (simulated)	2663 (simulated)	Flatband	5
		0.6 (measured)	140 (measured)		
*Opt*. *Lett*. 2025, 50, 2145 [37].	Simulated	0.89	30	Flatband	5
*Sci*. *Adv*. 2025, 11 (15), eads9562 [38].	Measured	0.98	2100	Non‐flatband	None
*Sci*. *Adv*. 2025, 11, eadw0986 [39].	Simulated/measured	1 (simulated)	7600 (simulated)	Flatband	90
		0.8 (measured)	200 (measured)		
*Advanced Photonics*, 2025, 7. 5: 056,008 [40].	Simulated	1 (simulated)	20,000 (simulated)	Flatband	19.87
		0.4 (measured)	1500 (measured)		
Our work	Simulated	0.91	20,000	Flatband	4.58

## 3 Conclusion

In summary, by tuning the interaction between four counterpropagating guided modes in a chiral measurface, we theoretically propose and numerically demonstrate nondispersive chiral BICs. In contrast to conventional BICs, we verify this nondispersive nature can sustain an effective light‐field confinement, enabling a robust chiral resonance with a high *Q*‐factor (> 20,000) and efficient third‐harmonic generation (conversion efficiency up to 2.3 × 10^−3^ under a pump intensity of 1 MW/cm^−2^) across a wide incident angular range. Our findings establish a new paradigm for developing high‐performance, miniaturized chiral, and nonlinear photonic devices with wide‐angle operation.

## Author Contributions

**Lei Huang:** funding acquisition, software, validation, methodology, conceptualization, writing – review and editing, visualization, supervision. **Weiqing Wang:** validation, software, writing – original draft, visualization. **Han Shen:** software. **Jing Chen:** methodology. **Shifeng Li:** validation, formal analysis. **Biao Liu:** conceptualization, methodology. **Fuhuan Shen:** conceptualization, supervision.

## Conflicts of Interest

The authors declare no conflicts of interest.

## Supporting information


Supporting Information S1

## Data Availability

The data that support the findings of this study are available from the corresponding author upon reasonable request.
