# Supplementary material for: Robust Chiral High‐Q Response Driven by Quasi‐Bound Flatband in the Continuum
Source: Nanophotonics. 2026 Jul 21;15(14):e70218. doi: 10.1002/nap2.70218 (PMC13642062; doi:10.1002/nap2.70218)
Supplement: Supplementary file 1 [file NAP2-15-e70218-s001.docx]

Supporting Information for

Robust chiral high-*Q* response driven by chiral quasi-bound flatband in the continuum

Lei Huang^1,2,4,^*^,^, Weiqing Wang^1^, Han Shen^1^, Jing Chen^1,^*, Shifeng Li^2^, Biao Liu^3^ and Fuhuan Shen^4^

*^1^State Key Laboratory of Flexible Electronics, College of Chemistry and Life Sciences, Nanjing University of Posts and Telecommunications, Nanjing 210023, P. R. China*

*^2^National Laboratory of Solid State Microstructures, College of Engineering and Applied Sciences, Nanjing University, Nanjing 210093, P. R. China*

*^3^School of Physics, Central South University, Changsha, Hunan 410083, P. R. China*

*^4^Division of Physics and Applied Physics, School of Physical and Mathematical Sciences, Nanyang Technological University, Singapore, Singapore*

*Correspondence: [iamleihuang@njupt.edu.cn](mailto:iamleihuang@njupt.edu.cn); [jchen@njupt.edu.cn](mailto:jchen@njupt.edu.cn)

**Note 1. Evolution of topological charger with asymmetry parameter**

The far-field eigenpolarization maps with the asymmetry parameter *α* = 0 for mode 2 are calculated in the inset of Figure S1a. An obvious polarization singularity (*V*-point) emerges at the *Γ* point, confirming that mode 2 is an SP-BIC. However, when we introduce the symmetry broken into the structure, the *V* point will split into two *C* points, which distance deviates from the *Γ* point, thus a chiral quasi-BIC with nonvanishing helicity is observed at the *Γ* point, as demonstrated by the far-field eigenpolarization maps in Figure S1b and c. Consequently, the mode emerging at the *Γ* point with nonvanishing helicity under *α* = 10 nm is more accurately described as a chiral quasi-BIC rather than a chiral BIC.

**Figure S1.** (a)-(c) show the schematic diagram of the chiral flatband metasurface with different asymmetry parameters. (d)-(f) the calculated far-field eigen-polarization maps for the three asymmetry parameters. The topological charges are splitting into two chiral topological charge with the introduction of asymmetry parameters.

**Note 2.** **Evidence of chiral flatband for mode 2**

To verify the wide-angle chiral response of the flatband mode, we calculated the circular dichroism (CD) and transmission spectra for four representative incident angles, i.e., sin(θ) = −0.08, −0.04, +0.04, and +0.08. In all cases, the resonance wavelength remains nearly fixed at ~1031 nm, confirming the dispersionless nature. Furthermore, the peak CD value exceeds 0.6 with a *Q*-factor > 20,000 across the entire angular range, demonstrating its robust performance.

**Figure S2.** (a)-(d) Transmission spectra of the flatband BICs at four representative incident angles with sin(*θ*) = −0.08, −0.04, 0, +0.04, and +0.08, respectively.
